# Effectiveness of cyclosporine as a treatment for steroid-resistant Cronkhite-Canada syndrome; two case reports

**DOI:** 10.1186/s12876-016-0541-1

**Published:** 2016-10-06

**Authors:** Kohei Yamakawa, Takuya Yoshino, Kotaro Watanabe, Koichiro Kawano, Akira Kurita, Naomi Matsuzaki, Yoshiaki Yuba, Shujiro Yazumi

**Affiliations:** 1 Division of Gastroenterology and Hepatology, Digestive Disease Center, Kitano Hospital, 2-4-20 Ohgimachi, Kita-ku, Osaka 530-8480 Japan; 2 Department of Pathology, Kitano Hospital, 2-4-20 Ohgimachi, Kita-ku, Osaka 530-8480 Japan

**Keywords:** Cronkhite-Canada syndrome, Cyclosporine, Steroid-resistance, Intestinal polyposis, Case report

## Abstract

**Background:**

Cronkhite-Canada syndrome (CCS) is a rare non-inherited disorder, characterized by gastrointestinal polyposis and ectodermal changes. The pathophysiology remains unclear. Treatment with corticosteroids is considered the mainstay treatment because of its high efficacy. However, some patients have steroid-resistant CCS. The therapeutic strategy for steroid-resistant CCS is not yet established. We report two cases with steroid-resistant CCS that were effectively treated with cyclosporine (CyA). We evaluated the therapeutic strategy for steroid-resistant CCS based on reviews of previous reports.

**Case presentation:**

Our patients with CCS were first treated with prednisolone. No clinical response was noted, and treatment with CyA was initiated. After beginning CyA treatment, both clinical symptoms and polyposis markedly improved. Up to the present, 55 cases of CCS treated with corticosteroids and their response were reported. Out of the 57 patients, including our 2 cases, 9 (16 %) did not respond clinically to corticosteroids. In 7 of the 9 steroid-resistant cases, the prognosis after corticosteroids treatment was described. In 5 of the 7 steroid-resistant cases, immunosuppressive treatments induced remission. In 4 of these 5 cases, moreover, the key drug of treatments was calcineurin inhibitor.

**Conclusions:**

Treatment with calcineurin inhibitor, such as CyA, could be a potential option for steroid-resistant CCS.

**Electronic supplementary material:**

The online version of this article (doi:10.1186/s12876-016-0541-1) contains supplementary material, which is available to authorized users.

## Background

Cronkhite-Canada syndrome (CCS), first reported by Cronkhite and Canada in 1955 [1], is a rare non-inherited disorder characterized by gastrointestinal polyposis accompanied by malabsorption and ectodermal changes, such as alopecia, onychodystrophy, and hyperpigmentation.

The pathophysiology of CCS remains unclear, despite many previous reports [1–5]. Because corticosteroid treatment for CCS is reported to be highly effective, it is considered the mainstay treatment for CCS. A recent study demonstrated the expression of autoimmune-related IgG4 antibody in CCS polyps [2]. The histological findings and response to immunosuppressive treatments suggested that immune response played an important role in the pathophysiology of CCS, and that a part of CCS might be one of IgG4-related disease. Although approximately 90 % of patients with CCS respond to corticosteroids, however, the remaining patients are refractory to corticosteroid treatment [2]. The therapeutic strategy for steroid-resistant CCS is not yet established. Here we report two cases with steroid-resistant CCS that were effectively treated with cyclosporine (CyA).

## Case presentation

### Case 1

**Fig. 1:**
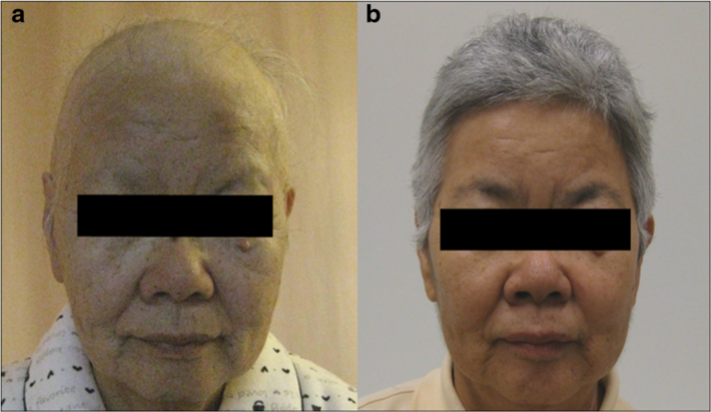
Face photos in Case 1. **a** Alopecia of not only the scalp, but also the eyebrows, was observed on admission. **b** Alopecia improved markedly after treatment with CyA

**Fig. 2:**
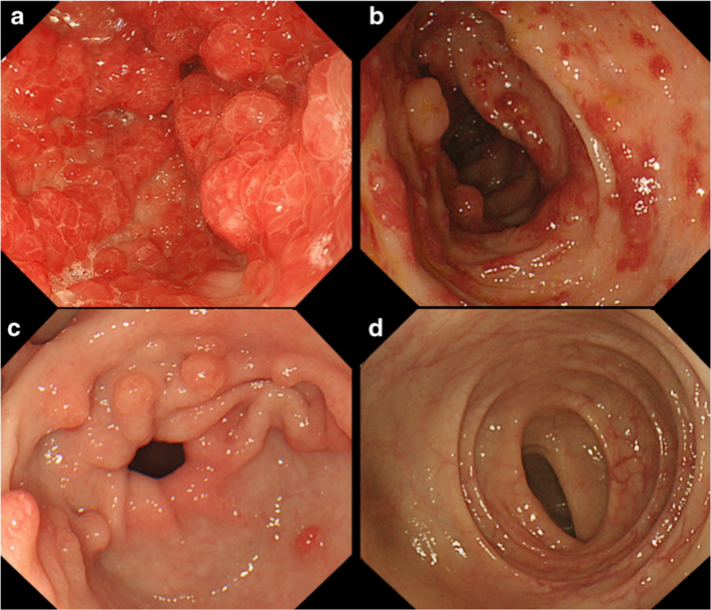
Endoscopic images in Case 1. The initial gastrointestinal endoscopy showed multiple reddish inflammatory polyps and edematous adjacent mucosa in the stomach (**a**) and colon (**b**). The endoscopy 18 months after treatment with CyA revealed marked improvement in many polyps in the stomach (**c**) and colon (**d**)

**Fig. 3:**
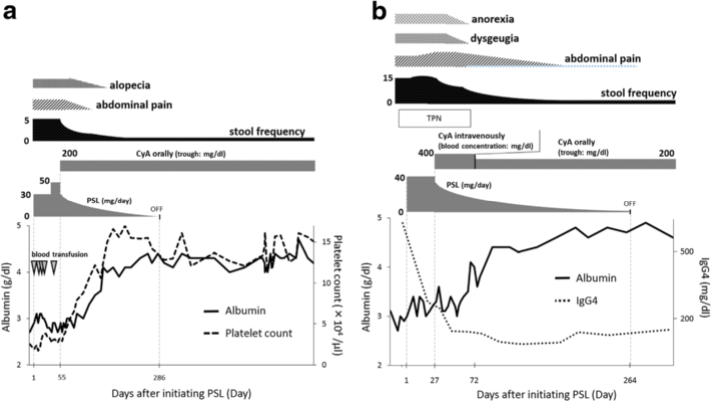
Clinical course in Case 1 and Case 2. **a** In Case 1, treatment with PSL at a daily dose of 0.6 mg/kg (30 mg) was initiated. Because of no clinical response to PSL 0.6 mg/kg, the dose of PSL was increased to at a daily dose of 1.0 mg/kg (50 mg). Because intensifying the dose of PSL wasn’t effective, CyA treatment was induced. After beginning CyA treatment, her symptoms and laboratory data, including platelet count, markedly improved, and blood transfusion was no longer needed. **b** In Case 2 with the high serum IgG4 level, treatment with PSL at a daily dose of 0.6 mg/kg (40 mg) and total parenteral nutrition was initiated. After initiating PSL treatment, the serum IgG4 level decreased. However, clinical symptoms were exacerbated and nutrition condition didn’t improve. Therefore, CyA treatment was induced. After beginning CyA treatment, his symptoms and laboratory data markedly improved, and CyA treatment was continued orally at a trough level of 200 ng/ml. After beginning CyA treatment, the serum IgG4 level decreased from 638 mg/dl to 97.0 mg/dl

A 75-year-old Japanese woman was admitted to our hospital with watery diarrhea and dysgeusia for two months, and alopecia for three weeks (Fig. 1a). Laboratory findings included hypoproteinemia (albumin: 2.9 g/dl, cholinesterase: 186 U/l, total cholesterol: 117 mg/dl) and pancytopenia (hemoglobin: 6.6 g/dl, platelet count: 1.9 × 10^4^ /μl, white blood cell count: 2700 /μl). The serum concentration of IgG and IgG4 (IgG 626 mg/dl, IgG4 43.5 mg/dl) was normal. Endoscopic findings revealed multiple reddish inflammatory polyps and edematous adjacent mucosa in the stomach, duodenum, terminal ileum, and colon (Fig. 2a and b). Histologic findings of polyps revealed prominent cystic dilation of the crypts and expanded inflamed lamina propria, showing few IgG4-positive plasma cells in the polyps. Moreover, bone marrow biopsy showed hypercellularity, 0.3 % of blast cells and a normal karyotype. Based on these clinical findings, CCS concomitant with myelodysplastic syndrome (MDS) was diagnosed. MDS was classified as refractory anemia according to French American British classification, and the International Prognostic Scoring System score was low. Despite treatment with prednisolone (PSL) at a daily dose of 0.6 mg/kg (30 mg) for 4 weeks, no clinical response was noted. Although the daily dose of PSL was increased to 1.0 mg/kg (50 mg), any clinical symptoms didn’t improve. This patient was regarded as steroid-resistant CCS who had ongoing active disease despite continuous treatment with systemic corticosteroids referring to previous report of steroid-refractory inflammatory bowel disease [6]. Considering the efficacy of CyA for MDS, CyA treatment was initiated orally at a trough concentration of approximately 200 ng/ml. After beginning CyA treatment, her symptoms and laboratory data, including pancytopenia, improved (Figs. 1b and 3a). PSL was gradually tapering and discontinued at 9.5 months after initiating PSL treatment. Endoscopic findings indicated the disappearance of several gastrointestinal polyps 18 months after beginning CyA treatment (Fig. 2c and d). For 52 months with CyA, there was no recurrence of symptoms, hypoproteinemia and polyps, and no incidence of gastrointestinal cancer.

### Case 2

**Fig. 4:**
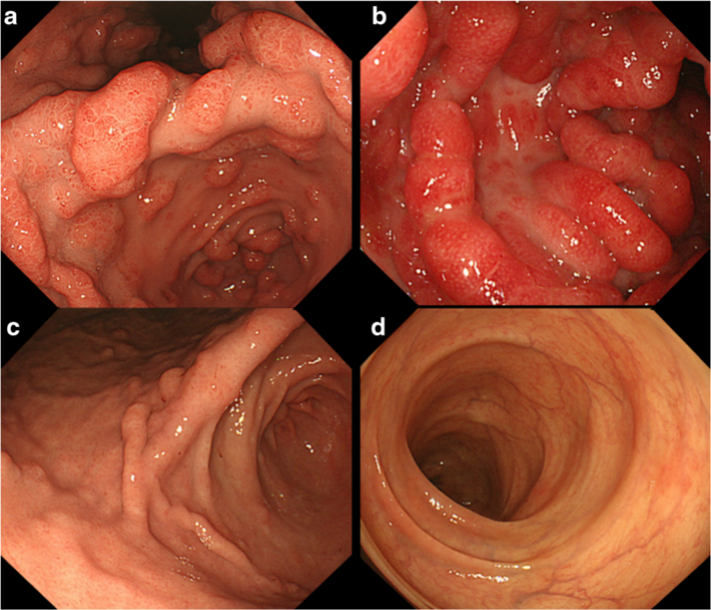
Endoscopic images in Case 2. The initial gastrointestinal endoscopy revealed numerous polyps in the stomach (**a**) and colon (**b**). The number of gastric (**c**) and colon (**d**) polyps was markedly reduced 8 months after initiating CyA treatment

**Fig. 5:**
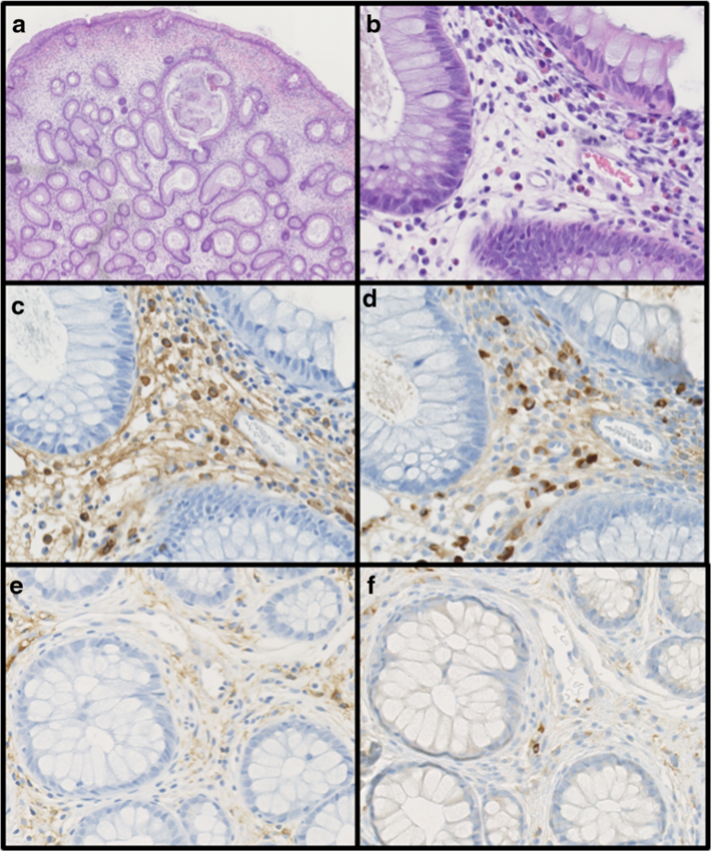
Histologic findings of the colonic polyp biopsy specimen in Case 2. Histologic findings demonstrated the prominent cystic dilation of the crypts (**a**) (hematoxylin and eosin stain; magnification × 40). Moreover, a mixed inflammatory infiltrate with prominent eosinophils was found (**b**) (hematoxylin and eosin stain; magnification × 400). IgG-positive plasma cells numbered 60/HPF (**c**) (IgG-immunostaining; magnification × 400). In the same field, the high number of IgG4-positive plasma cells was found (50 labeled cells per HPF) (83 % IgG4/IgG plasma cells) (**d**) (IgG4-immunostaining; magnification × 400). Few IgG- and IgG4-positive plasma cells were found after treatment with CyA (**e**) (IgG-immunostaining; magnification × 400), (f) (IgG4-immunostaining; magnification × 400)

A 50-year-old Japanese man presented with watery diarrhea and hyperpigmentation for two months, dysgeusia and abdominal pain for two weeks, and a 10-kg weight loss. Physical examination revealed onychatrophia and hyperpigmentation. Laboratory data revealed hypoproteinemia (albumin: 2.7 g/dl, cholinesterase: 128 U/l, total cholesterol: 108 mg/dl) and elevated IgG and IgG4 concentrations (IgG 1630 mg/dl, IgG4 638 mg/dl). Endoscopic findings revealed numerous polyps in the stomach, duodenum, and colon (Fig. 4a and b). Histologic findings of the polyps indicated prominent cystic dilated crypts and mixed inflammatory infiltrate with eosinophils. Moreover, IgG4-positive plasma cells were increased in the lamina propria (50 labeled cells per high-power field [HPF]; Fig. 5a, b, c and d). CCS was diagnosed based on those findings. Treated with PSL at a daily dose of 0.6 mg/kg (40 mg) for 4 weeks, the serum IgG4 concentration markedly decreased. However, his abdominal symptoms worsened. Therefore, CyA treatment was initiated intravenously at a blood concentration of 400 ng/ml and PSL was tapered 4 weeks after initiating PSL treatment referring to previous report [7]. After beginning CyA treatment, his symptoms and laboratory data markedly improved (Fig. 3b). CyA treatment was continued orally at a trough concentration of approximately 200 ng/ml, and PSL was discontinued at 8.8 months after initiating PSL treatment. Finally, the serum IgG4 concentration decreased from 638 mg/dl to 97.0 mg/dl. Endoscopic findings revealed a reduction in gastrointestinal polyps 8 months after initiating CyA treatment (Fig. 4c and d). Moreover, few IgG4-positive plasma cells were found in lamina propria of the polyps and adjacent mucosa (Fig. 5e and f).

## Discussion

CCS is a progressive disease with various courses and a 5-year mortality rate was greater than 50 % [3]. To improve the poor prognosis of CCS, various treatments have been proposed, such as nutritional support, antibiotics, corticosteroids, immunomodulators, biologics, and surgery [4]. Corticosteroids are reported to be highly effective for treating CCS [2, 5]. The prognosis after treatment with corticosteroids, however, is unknown. In our cases, no clinical response was found despite corticosteroids treatment, although the serum IgG4 concentration decreased after initiating corticosteroids treatment in Case 2. CyA treatment could dramatically improve clinical symptoms and endoscopic findings, and maintain remission for a long time after discontinuing corticosteroids. Therefore, CyA treatment was considered effective for our both cases, although the possibility of the delayed response to steroid could not be denied completely.

A PubMed review of the English literature revealed 55 cases of CCS treated with corticosteroids for which the response was clearly described. Of the 57 patients, including our 2 cases, a symptomatic response was reported for 48 (84 %). On the other hand, the remaining 9 patients (16 %) did not respond clinically to corticosteroids. (Table 1) [2, 4, 7–11]. Among the 9 steroid-resistant cases, 7 cases described the clinical course after the treatment with corticosteroids and 2 cases didn’t. Out of these 7 patients, five could achieve clinical remission by immunosuppressive treatments (four patients: calcineurin inhibitor, one patient: infliximab), one received colectomy, and last one died without additional treatment.

**Table 1:** Case reports regarding steroid-resistant CCS

Case	Age /Sex	Previoius Treatment	Next Treatment	Mentenance treatment	Follow-up duration (month)	Prognosis	Reference /year
1	57/M	Corticosteroids Azathioprine Ileocecal resection	Infliximab	Infliximab	36	Symptom: Remission Polyp: Remission	[4] /2014
2	71/M	Prednisolone	Cyclosporine	Azathioprine	23	Symptom: Remission Polyp: ND	[7] /2014
3	42/M	Prednisolone	Colectomy	nothing	6	Symptom: Remission Polyp: Remission	[8] /2013
4	ND	Prednisolone	ECP	ND	ND	Symptom: ND Polyp: ND	[2] /2012
5	50/M	Prednisolone	Colectomy	ND	ND	Symptom: ND Polyp: ND	[9] /2012
6	80/M	Prednisolone	Nothing			Dead	[10] /2008
7	44/M	Prednisolone	Tacrolimus Azathioprine	Azathioprine	18	Symptom: Remission Polyp: Remission	[11] /2006
8	75/F	Prednisolone	Cyclosporine	Cyclosporine	52	Symptom: Remission Polyp: Remission	Case1
9	50/M	Prednisolone	Cyclosporine	Cyclosporine	8	Symptom: Remission Polyp: Remission	Case2

*ND* not described, *ECP* endoscopic cyclophotocoagulation

An autoimmune mechanism has been more strongly suggested to underlie the pathophysiology of CCS since a recent study showed that IgG4-positive plasma cells infiltrated the lamina propria in the polyps of patients with CCS (positive IgG4 immunostaining [>5 cells/HPF] in 22 (52 %) of 42 polyps from 13 CCS patients) [2]. A basic research reported that activation of Toll-like receptors (TLRs) in basophils of patients with IgG4-related diseases induced a large amount of IgG4 by B cells, this enhancement of IgG4 production was associated with B cell activating factor (BAFF) and IL-13 [12]. In our Case 2, the high serum concentrations of IgG4 and the high number of IgG4-positive plasma cells in gastrointestinal polyps were detected before CyA treatments. Additionally, both the serum concentrations of IgG4 and the number of IgG4-positive plasma cells in gastrointestinal polyps could improve after PSL and CyA treatments. Previous and our data suggest that a part of CCS would be the IgG4-related disease due to IgG4 producing B cells with BAFF and IL-13 production through immune response, particularly innate immune response.

Although those findings regarding IgG4 expression weren’t found in our Case 1, concomitant MDS was observed in our Case 1. Previously, Suzuki et al. also reported a CCS case associated with MDS [13]. The incidence rate of MDS in CCS patients was unclear, whereas several retrospective studies and a recent cohort study have demonstrated the high incidence rate (10–28 %) of autoimmune diseases, such as hypothyroidism and rheumatoid arthritis, in patients with MDS [14–17]. Thus, there is the strong association between autoimmune diseases and MDS. Surprisingly, this cohort study reported that MDS patients with autoimmune diseases had significantly better clinical outcome including overall survival rate and leukemia transformation rate compared to those without autoimmune diseases [17]. In our Case 1, MDS concomitant with CCS was classified as low risk based on the International Prognostic Scoring System score, and the prognosis was expected to be good. These data also suggested that CCS might be autoimmune disease. Furthermore, aggressive immunosuppressive treatments in MDS with autoimmune diseases were reported to be effective for not only controlling autoimmune phenomena but also leading to hematological response [14]. Taking these data into account, immunosuppressive treatments, particularly CyA, would be reasonable for CCS concomitant with MDS, like our Case 1.

According to those data, the immunological disorder would be involving in the pathophysiology of CCS. Therefore, immunosuppressive treatments with calcineurin inhibitor, such as CyA, should be considered as a treatment for patients with steroid-resistant CCS, although our gastroenterologists need pay attention to the risk of adverse events, such as severe infection and malignancies.

## Conclusion

We report that CyA treatment was effective for two patients with steroid-resistant CCS, and suggest that the treatment with calcineurin inhibitor, such as CyA, could be a therapeutic option for steroid-resistant CCS.

## Additional file

Additional file 1: CARE Checklist – 2016: Information for writing a case report. (DOCX 613 kb)

## Abbreviations

BAFF: B cell activating factor
CCS: Cronkhite-Canada syndrome
CyA: Cyclosporine
MDS: Myelodysplastic syndrome
PSL: Prednisolone
TLRs: Toll-like receptors.
