# Supplementary material for: Effectiveness of cyclosporine as a treatment for steroid-resistant Cronkhite-Canada syndrome; two case reports
Source: BMC Gastroenterol. 2016 Oct 6;16:123. doi: 10.1186/s12876-016-0541-1 (PMC5053148; doi:10.1186/s12876-016-0541-1)
Supplement: Additional file 1: — CARE Checklist – 2016: Information for writing a case report. (DOCX 613 kb) [file 12876_2016_541_MOESM1_ESM.docx]

**CARE Checklist – 2016: Information for writing a case report**

**Topic Item Checklist item description Line/Page**

**Title 1** The words “case report” should be in the title along with the area of focus _1-2/1_

**Key Words 2** Four to seven key words—include “case report” as one of the key words _18/3_

**Abstract 3a** Background: What does this case report add to the medical literature? _2-7/3_

**3b** Case summary: chief complaint, diagnoses, interventions, and outcomes 8-14/3_

**3c** Conclusion: What is the main “take-away” lesson from this case? 15-16/3_

**Introduction 4** The current standard of care and contributions of this case—with references (1-2 paragraphs) 1-13/4

**Timeline 5** Information from this case report organized into a timeline (table or figure) Figures 3AB

**Patient Information 6a** De-identified demographic and other patient or client specific information __ N/A _

**6b** Chief complaint—what prompted this visit? 17-18/4, 15-16/5

**6c** Relevant history including past interventions and outcomes __N/A__

**Physical Exam 7** Relevant physical examination findings _16-17/5

**Diagnostic 8a** Evaluations such as surveys, laboratory testing, imaging, etc. 18-25/4, 17-22/5

**Assessment 8b** Diagnostic reasoning including other diagnoses considered and challenges 17-25/4, 15-22/5

**8c** Consider tables or figures linking assessment, diagnoses and interventions Figures 1,2,4,5

**8d** Prognostic characteristics where applicable 25/4-2/5

**Interventions 9a** Types such as life-style recommendations, treatments, medications, surgery 2/5, 23/5

**9b** Intervention administration such as dosage, frequency and duration 2-4/5, 23-24/5

**9c** Note changes in intervention with explanation 4-9/5, 23/5-3/6

**9d** Other concurrent interventions __N/A__

**Follow-up and 10a** Clinician assessment (and patient or client assessed outcomes when appropriate) 9-12/5, 3-6/6

**Outcomes 10b** Important follow-up diagnostic evaluations 9-12/5, 3-6/6

**10c** Assessment of intervention adherence and tolerability, including adverse events __ N/A__

**Discussion 11a** Strengths and limitations in your approach to this case 13-17/6

**11b** Specify how this case report informs practice or Clinical Practice Guidelines (CPG) 24/7-1/8_

**11c** How does this case report suggest a testable hypothesis? 25/6-23/7

**11d** Conclusions and rationale _2-5/8 _

**Patient Perspective 12** When appropriate include the assessment of the patient or client on this episode of care 25/6-23/7

**Informed Consent 13** Informed consent from the person who is the subject of this case report is required by most journals _10-12/9_

**Additional Information 14** Acknowledgement section; Competing Interests; IRB approval when required 6-8,14-15/9
